# *GhL1L1* affects cell fate specification by regulating GhPIN1‐mediated auxin distribution

**DOI:** 10.1111/pbi.12947

**Published:** 2018-05-31

**Authors:** Jiao Xu, Xiyan Yang, Baoqi Li, Lin Chen, Ling Min, Xianlong Zhang

**Affiliations:** ^1^ National Key Laboratory of Crop Genetic Improvement Huazhong Agricultural University Wuhan Hubei China; ^2^ College of Plant Science & Technology Huazhong Agricultural University Wuhan Hubei China

**Keywords:** cotton, auxin, cell fate, GhPIN1, GhPP2AA2, GhL1L1

## Abstract

Auxin is as an efficient initiator and regulator of cell fate during somatic embryogenesis (SE), but the molecular mechanisms and regulating networks of this process are not well understood. In this report, we analysed SE process induced by Leafy cotyledon1‐like 1 (*GhL1L1*), a NF‐YB subfamily gene specifically expressed in embryonic tissues in cotton. We also identified the target gene of *GhL1L1*, and its role in auxin distribution and cell fate specification during embryonic development was analysed. Overexpression of *GhL1L1* accelerated embryonic cell formation, associated with an increased concentration of IAA in embryogenic calluses (ECs) and in the shoot apical meristem, corresponding to altered expression of the auxin transport gene *GhPIN1*. By contrast, *GhL1L1*‐deficient explants showed retarded embryonic cell formation, and the concentration of IAA was decreased in *GhL1L1*‐deficient ECs. Disruption of auxin distribution accelerated the specification of embryonic cell fate together with regulation of GhPIN1. Furthermore, we showed that *PHOSPHATASE 2AA2* (*GhPP2AA2*) was activated by GhL1L1 through targeting the G‐box of its promoter, hence regulating the activity of GhPIN1 protein. Our results indicate that GhL1L1 functions as a key regulator in auxin distribution to regulate cell fate specification in cotton and contribute to the understanding of the complex process of SE in plant species.

## Introduction

Somatic embryogenesis (SE) is a process of asexual reproduction in plants in which somatic cells undergo differentiation and redifferentiation to form embryos. It resembles zygotic embryogenesis, whereby globular‐, torpedo‐ and cotyledonary‐stage embryos are formed. SE consists of direct SE and indirect SE. In former, embryoids are produced from the explant directly during *in vitro* culture, while indirect SE is more complex whereby somatic cell dedifferentiation leads to callus formation and then redifferentiation into embryogenic calluses (ECs) and somatic embryos. Cotton undergoes indirect SE, and the process can be divided into several stages, including the dedifferentiation of cotton somatic cells and transition from nonembryogenic calluses (NEC) to ECs, followed by the development of somatic embryos (Yang *et al*., 2012).

As an excellent natural provider of fibre, cotton needs a reproducible and highly efficient plant regeneration scheme for transgenic research and genetic engineering. The morphological and molecular mechanisms of SE have been studied in cotton in our laboratory (Jin *et al*., 2014; Yang *et al*., 2012; Zhou *et al*., 2016). *GhHmgB3*‐deficient hypocotyls dedifferentiate more rapidly but fail to differentiate into ECs (Hu *et al*., 2011). By contrast, overexpression of *GhCKI* prevents EC and plant regeneration by blocking the transition from NECs to ECs (Min *et al*., 2015). However, the precise mechanisms of gene regulation during cotton SE have not been elucidated.

Transcription factors are considered to play important roles during the process of SE, and the *Leafy cotyledon* (*LEC*) genes are major regulators of embryo development and cellular differentiation (Kwong *et al*., 2003; Lee *et al*., 2003; Lotan *et al*., 1998). Mutations of *LEC1* result in defective embryo maturation (Lotan *et al*., 1998; Meinke, 1992; Meinke *et al*., 1994). *LEC2*,* FUS3* and *ABI3* have been considered as marker genes during embryogenic cell formation (Braybrook and Harada, 2008; Gazzarrini *et al*., 2004; Wang and Perry, 2013). *LEC* genes are found to regulate auxin homeostasis during embryogenesis (Braybrook *et al*., 2006; Gazzarrini *et al*., 2004; Kagaya *et al*., 2005; Stone *et al*., 2008), and *LEC1* and *LEC1‐like* (*L1L*) are partially functionally redundant (Kwong *et al*., 2003; Yamamoto *et al*., 2009). LEC1 acts as a coactivator of PIF4 to co‐regulate etiolation‐related genes during postembryonic growth in the dark (Huang *et al*., 2015). *LEC2* is considered to regulate *YUCCA4*, which encodes an auxin biosynthetic enzyme, required for somatic embryo formation (Braybrook *et al*., 2006; Stone *et al*., 2001, 2008). *FUS3* interacts with *LEC2* to promote auxin biosynthesis (Tang *et al*., 2017). Disruption of auxin homeostasis by *GhCKI* overexpression, which might act downstream of *GhLEC1*, leads to defective embryogenesis (Min *et al*., 2015).

Auxin regulation during plant SE has been well documented in model systems (Kim *et al*., 2000; Komamine *et al*., 2005; Quint and Gray, 2006). Auxin gradients modulate the response and transduction of the auxin signal to regulate the expression of genes during phase changes between cell division and cell differentiation during SE (Chugh and Khurana, 2002; Jimenez, 2005; Rose and Nolan, 2006; Yang and Zhang, 2010; Yang *et al*., 2012). Auxin plays an important role in *WUS* expression, which is essential for embryonic stem cell fate determination during SE in *Arabidopsis* (Mayer *et al*., 1998; Su *et al*., 2009). Research has also demonstrated that the interactions between auxin, ethylene, gibberellic acid and stress response regulate SE (Wang *et al*., 2018; Zheng *et al*., 2013, 2016; Zhou *et al*., 2016).

Auxin gradients are established by local auxin biosynthesis, degradation or polar auxin transport (Vanneste and Friml, 2009; Wabnik *et al*., 2013). *DR5*, a highly active synthetic auxin response element (*AuxRE*) reporter gene, reveals the distribution of auxin in tissues and cells (Ulmasov *et al*., 1997; Zhang *et al*., 2016). Auxin biosynthesis spatially and temporally regulated by YUCs is an essential source of auxin during embryogenesis, floral development and vascular patterning (Cheng *et al*., 2006; Zhao, 2010). It has been demonstrated that PIN‐dependent auxin transport and the auxin gradient play important roles during the formation of the apical‐basal embryo axis (Friml *et al*., 2003). The *Arabidopsis PIN* gene family consists of eight members, and auxin efflux in the embryo is mediated by PIN1, PIN4 and PIN7 (Friml *et al*., 2003; Paponov *et al*., 2005). PIN1 is expressed in proembryogenic cells in a nonpolar manner during the early developmental stages and then becomes polarized to the basal side of provascular cells during the early globular stage (Friml *et al*., 2003; Steinmann *et al*., 1999). The localization of PIN1 changes from the apical cells in 16‐cell stage embryos to the basal side in early heart stage embryos (Grunewald and Friml, 2010; Guenot *et al*., 2012). Directional auxin movement depends on the phosphorylation status of the PINs, which affects their polar subcellular localization. PINOID kinase and PP2A phosphatase are important regulators of PIN targeting and so of auxin distribution (Friml *et al*., 2004; Michniewicz *et al*., 2007; Zhang *et al*., 2010). Moreover, some auxin polar transport inhibitors, such as 2,3,5‐triiodobenzoic acid (TIBA), can inhibit auxin transport by affecting the localization of PIN proteins and can be used to investigate the accumulation of auxin (Geldner *et al*., 2001; Klima *et al*., 2015).

In this study, a LEC1‐type gene, *GhL1L1,* was identified by RNA‐Seq as being expressed during cotton SE (Yang *et al*., 2012), and is specifically expressed in cotton embryonic tissues. Overexpression of *GhL1L1* reorganized cell patterning during cell dedifferentiation and accelerated cell fate specification during embryonic development, with a change in auxin homeostasis. Disruption of *GhL1L1* expression resulted in the opposite phenotype. We propose that *GhL1L1* mediates auxin distribution to regulate cell fate specification by initiating the interaction between GhPP2AA2 and GhPIN1 proteins through binding to the cis‐element within the promoter of *GhPP2AA2*.

## Results

### *GhL1L1* is specifically expressed in cotton embryonic tissues

**Figure 1:**
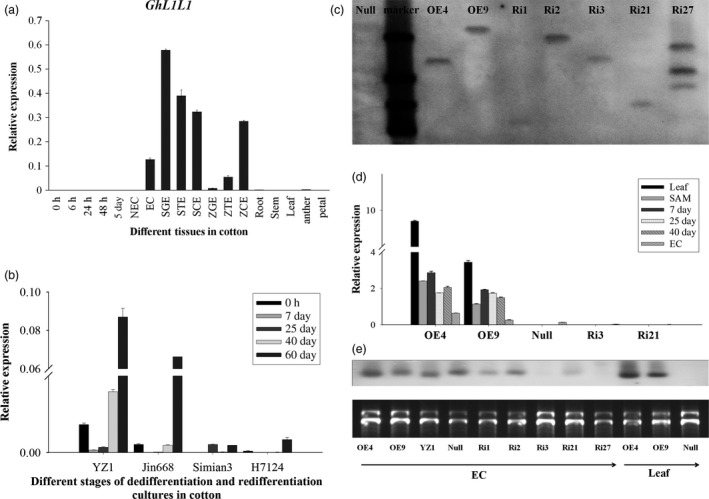
Expression analysis of *GhL1L1*. (a) qRT‐PCR analysis of *GhL1L1* (0, 6, 24, 48 h and 5 days explants; NEC, nonembryogenic callus; EC, embryogenic callus; SGE, somatic globular embryo; STE, somatic torpedo embryo; SCE, somatic cotyledon embryo; ZGE, zygotic globular embryo; ZTE, zygotic torpedo embryos; ZCE, zygotic cotyledon embryo). (b) qRT‐PCR analysis of *GhL1L1* in YZ1, Jin668, Simian3 and H7124. (c) Southern blotting of transgenic cotton plants, OE4 and OE9 represent the overexpression lines, Ri1, Ri2 and Ri3 represent RNA interference of 3′ untranslated region lines, Ri21and Ri27 represent RNA interference of coding region lines. (d) qRT–PCR analysis of *GhL1L1* in different transgenic and *null* lines in leaf, shoot apical meristem (SAM), 7, 25, 40 days explants and ECs. (e) Northern blot analysis of *GhL1L1* in different transgenic and *null* lines in ECs and leaf. The data (in a, b and d) are shown as the mean ± SE (*n* = 3).

*GhL1L1* was identified during a RNA‐Seq profiling analysis during cotton SE (Yang *et al*., 2012). The 651 bp cDNA encodes a NF‐YB subfamily protein of 216 amino acids. GhL1L1 protein has a highly conserved central B domain (Figure S1). The transcript of *GhL1L1* specifically accumulates in ECs, somatic embryos [somatic globular embryos (SGEs), somatic torpedo embryos (STEs), somatic cotyledon embryos (SCEs)] and zygotic embryos [zygotic globular embryos (ZGEs), zygotic torpedo embryos (ZTEs), zygotic cotyledon embryos (ZCEs)], with the greatest abundance in SGEs. Expression of *GhL1L1* was very low in root, stem, leaf and other nonembryonic tissues, as well as in cells in the dedifferentiation stage (0, 6, 24, 48 h, 5 days and NECs) (Figure 1a). The expression of *GhL1L1* was also detected during the dedifferentiation and redifferentiation stages in four cotton varieties with different regeneration abilities. The highest expression levels were observed in YZ1, the genotype with the highest regeneration efficiency, followed by relatively high levels in Jin668, a genotype that regenerates with moderate efficiency. By contrast, expression levels were low in Simian3 and H7124, which are recalcitrant to regeneration in both *Gossypium hirsutum* and *Gossypium barbadense* (Figure 1b).

A total of 41 NF‐YB subfamily genes were identified genome‐wide in *G. hirsutum* (TM‐1) (Figure S2a). Phylogenetic analysis revealed six *LEC1*‐type genes in *G. hirsutum, GhL1L1A* (*Gh_A05G1515*), *GhL1L1D* (*Gh_D05G1686*), *GhL1L2A* (*Gh_A13G1116*), *GhL1L2D* (*Gh_D13G1387*), *GhL1L3A* (*Gh_A08G0216*) and *GhL1L3D* (*Gh_D08G0296*), among which A and D represent different copies that are distributed to the A and D subgenomes, respectively, and which encode similar proteins, with several nucleotide polymorphisms in the CDS regions of the A and D subgenomes.

The expression patterns of the 41 *NF‐YB* genes were analysed in *G. hirsutum* (TM‐1) using public data sets. The six *LEC1*‐type genes were specifically expressed in 20‐day seeds, with the most abundant expression observed for *GhL1L1D* (Gh_D05G1686). Some of the other *NF‐YB* genes were also expressed in other tissues (Figure S2b). Three B3 domain genes [*GhLEC2* (*Gh_A09G0695*), *GhFUS3* (*Gh_A07G2123*), *GhABI3* (*Gh_D07G1550*)] were also identified in upland cotton. All the *LEC1*‐type genes and B3 domain genes showed expression patterns specifically during cotton embryogenesis (Figure S3).

### *GhL1L1* positively regulates cell fate specification during cotton SE

To understand the function of *GhL1L1* in cotton, one overexpression and two RNAi vectors including the coding region and 3′ untranslated region of *GhL1L1* were constructed and transformed into *G. hirsutum* YZ1. Several single T‐DNA insertion lines were identified by Southern blot analysis and selected for further analysis (Figures 1c and S4). qRT‐PCR and northern blotting revealed that *GhL1L1* transcript accumulated in leaf, shoot apical meristem (SAM) and dedifferentiation stage explants, and was also high in ECs of the overexpression lines, but low levels accumulated in the RNAi lines (Figure 1d,e). We selected two overexpression lines (OE4, OE9, with higher expression levels in OE4 than in OE9), one 3′ untranslated region RNAi line (Ri3), one coding region RNAi line (Ri21) and a *null* line (a negative plant line isolated from the offspring of OE4) for further study.

**Figure 2:**
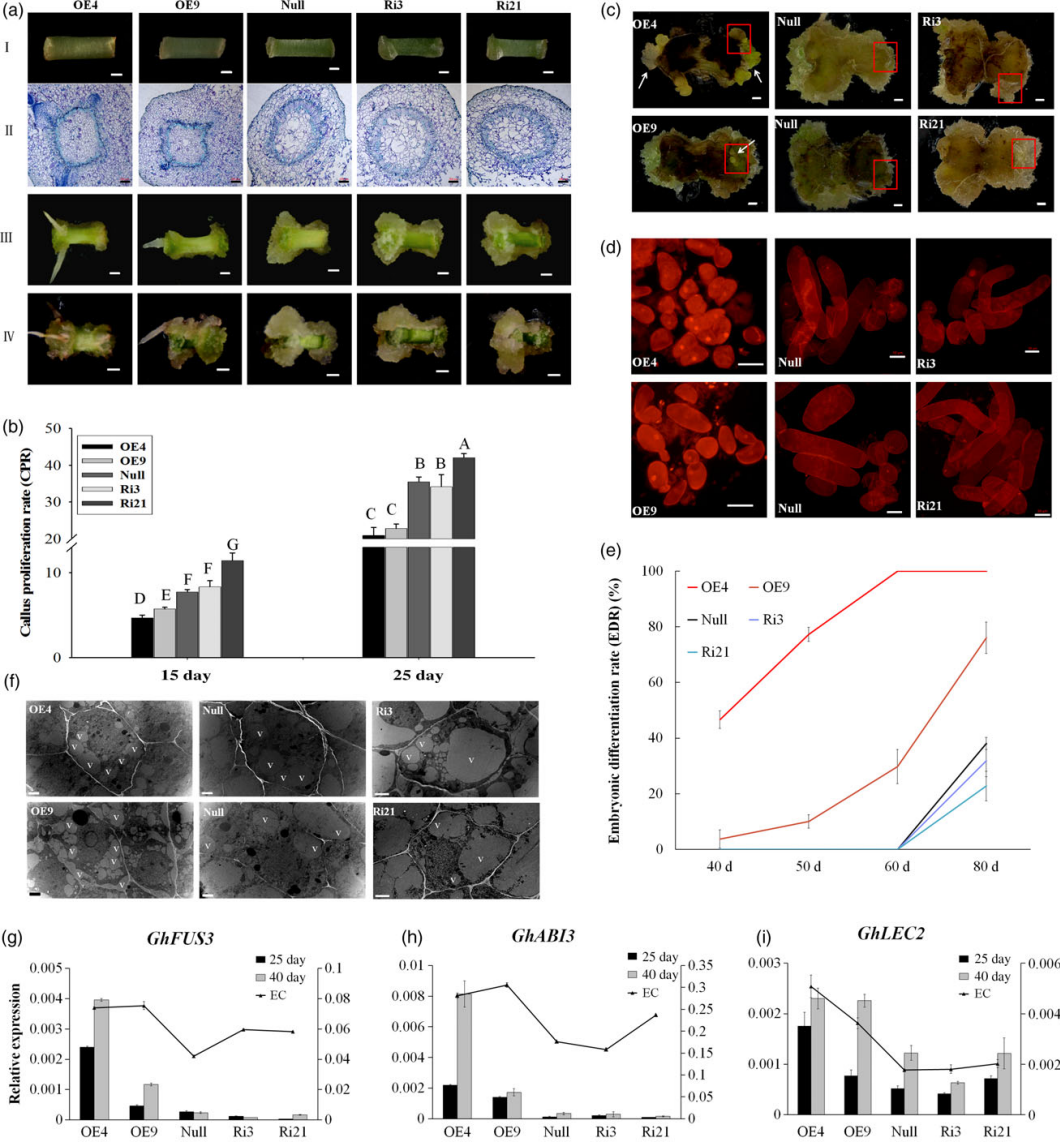
*GhL1L1* positively regulates cell fate specification during cotton SE. (a) The phenotypes of different *GhL1L1* transgenic and *null* lines at 7 (I, scale bars = 1 mm; II, scale bars = 200 μm), 15 (III, scale bars = 5 mm) and 25 days postinduction (IV, scale bars = 5 mm). (b) The callus proliferation rate (CPR) of different transgenic and *null* lines at 15 and 25 days post‐induction. Different capital letters denote significant differences by multiple comparisons using Statistix 8.0 software. (c) The ECs or embryos were observed for the overexpression line (OE4 and OE9) explants (white arrows) at 40 days postinduction, scale bars = 2.5 mm. (d) Cellular features of the calluses from red boxes of (c), scale bars = 50 μm. (e) The embryonic differentiation rate (EDR) of different transgenic lines and null at 40, 50, 60 and 80 days post‐induction. (f) The ECs of different transgenic lines and *null* scanned by transmission electron microscopy. v, vacuole. Scale bars = 2 μm. qRT–PCR analysis shows *GhFUS3* (g), *GhABI3* (h) and *GhLEC2* (i) in *GhL1L1* transgenic and *null* lines at 25 and 40 days post‐induction and ECs (embryogenic calluses from the corresponding transgenic lines and *null*). The data (in b, e, g, h and i) are shown as the mean ± SE (*n* = 3).

Calluses were induced from each transgenic lines (OE4, OE9, Ri3, Ri21) and the *null* on MSB medium *in vitro*. Seven days postinduction, calluses could be observed at the ends of *null* and RNAi line explants, while only a little expansion with adventitious roots was observed at the ends of OE4 and OE9 explants. Histological observation showed reorganized cell patterning from the cambium areas in OE4 and OE9, while vascular cells overproliferated in Ri3 and Ri21. During the development of SE, the polar growth was evident for Ri3 and Ri21 on 15 and 25 days postinduction. However, the explant ends of the overexpression lines (OE4 and OE9) showed little difference (Figure 2a). The callus proliferation rate (CPR) was then measured among those lines during the dedifferentiation stage. The results showed that overexpression of *GhL1L1* inhibited callus proliferation at both ends of the hypocotyls, while increased CPR was observed only in the RNAi line (Ri21) at all tested time points, compared with the *null* (Figure 2b). Therefore, we conclude that the expression of *GhL1L1* leads to a reorganization of the patterning of cambium cells and restricts uncontrolled callus proliferation.

Overexpression of *GhL1L1* promoted embryonic cell differentiation during cell culture, which accounted for an embryonic differentiation rate (EDR) of 46.6% in OE4 and 3.8% in OE9 with observable ECs at 40 days postinduction. Round small cells and a large nucleus were observed in cells of overexpression lines (Figure 2c,d and e). However, in *null* and RNAi lines no ECs were present, with large long cells and an unclear nucleus (Figure 2c,d). Some ECs were observed in OE4 at 25 days postinduction, while the *null* and RNAi lines produced none until at least 60 days postinduction, and were clearly visible at approximately 80 days postinduction. An EDR of less than 40% (*null*, 38%; Ri3, 31.9%; Ri21, 22.8%) were seen in *null* and RNAi lines, with more than 70% (OE4, 100%; OE9, 76%) in overexpression lines at the same time point (Figure 2e). Compared with *null*, many small vacuoles were observed in ECs of OE4 and OE9, while large vacuoles were present in ECs of Ri3 and Ri21 (Figure 2f).

*ABI3*,* FUS3* and *LEC2* are considered as marker genes during embryonic stem cell fate determination (Braybrook and Harada, 2008; Gazzarrini *et al*., 2004; Wang and Perry, 2013). *GhABI3* and *GhFUS3* were specifically expressed in embryonic cells (ECs and somatic embryos) (Figure S3d,e). The expression of *GhABI3* and *GhFUS3* could not be detected at 7 days postinduction, but the expression levels of the three genes were significantly up‐regulated in OE4 and OE9 compared with the RNAi lines and *null* at 25 and 40 days postinduction (Figure 2g–i). Some embryonic cells were present when overexpressing *GhL1L1* even at 25 days postinduction. These results suggest that overexpression of *GhL1L1* accelerates cell fate specification, while repression of *GhL1L1* retards embryonic cell differentiation.

### *GhL1L1* affects auxin accumulation and auxin distribution in cotton

**Figure 3:**
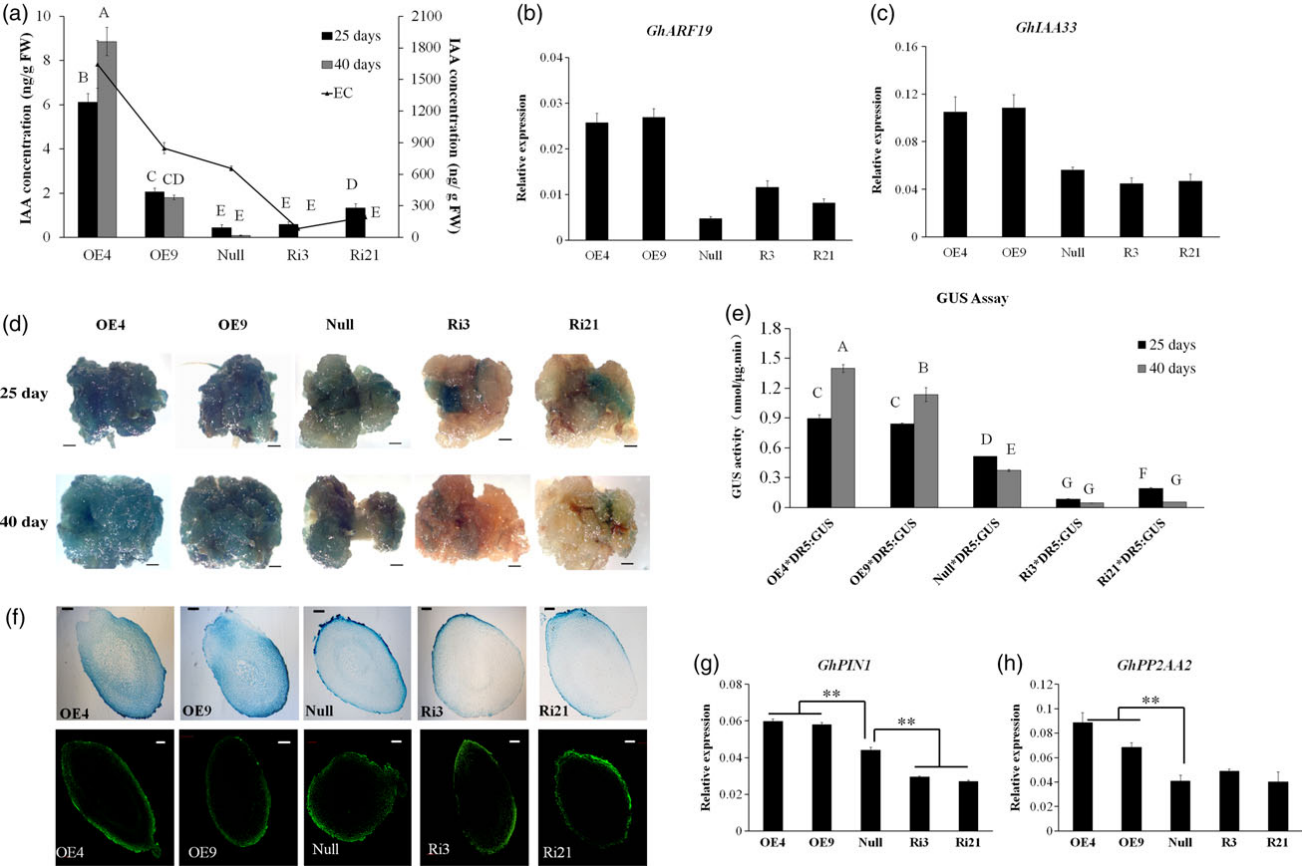
Auxin accumulation and distribution in *GhL1L1* transgenic and *null* lines during SE. (a) The IAA concentration in different transgenic and *null* lines at 25 and 40 days postinduction and ECs (embryogenic calluses from the corresponding transgenic and *null* lines). Expression analysis of the auxin response genes *GhARF19* (b) and *GhIAA33* (c) by qRT‐PCR. GUS expression (d) and GUS activity (e) of explants from five F_1_ hybrids (*OE4/DR5::GUS, OE9/DR5::GUS, Ri3/DR5::GUS, Ri21/DR5::GUS, null/DR5::GUS*) at 25 and 40 days postinduction, scale bars = 2.5 mm. Different capital letters denote significant differences by multiple comparisons using Statistix 8.0 software. (f) GUS staining (upper) and IAA immunofluorescence (below) of longitudinal section of torpedo embryos from *GhL1L1* transgenic and *null* lines, scale bars = 100 μm. Expression analysis of *GhPIN1* (g) and *GhPP2AA2* (h) in ECs of different transgenic and *null* lines. The data (in a, b, c, e, g and h) are shown as the mean + SE (*n* = 3). Statistical analyses were performed using Student's *t‐*test. **p <* *0.01.

Given the importance of auxin during cotton SE, the concentration of IAA in the different transgenic lines at 25 and 40 days postinduction and in ECs was measured by HPLC‐MS. After 25 and 40 days of induction, the IAA concentration of explants in the overexpression lines was higher than in the *null* and RNAi explants. Additionally, the concentration of endogenous IAA was increased in the *GhL1L1* overexpression ECs. By contrast, it was decreased in the ECs of RNAi lines compared with *null* (Figure 3a), and the transcription of two auxin response genes, *ARF19* and *IAA33*, was increased after overexpressing *GhL1L1* in ECs (Figure 3b,c).

To investigate the function of *GhL1L1* in auxin accumulation and distribution, *GhL1L1* transgenic lines containing *DR5::GUS* were generated to monitor auxin distribution. Explants of the hybrids were sampled for GUS staining and GUS activity at 25 and 40 days postinduction, respectively. Clear GUS staining was observed in the calluses of explants from the hybrids of *OE‐GhL1L1*/*DR5::GUS*, with only a little GUS staining in the hybrid of *null*/*DR5::GUS* and almost no GUS staining in *RNAi‐GhL1L1*/*DR5::GUS* (Figure 3d). As expected, GUS activity corresponded to GUS staining. GUS activity was much stronger in the calluses of explants from hybrids of *OE‐GhL1L1*/*DR5::GUS* and was lower in *RNAi‐GhL1L1*/*DR5::GUS* lines than the hybrid of *null*/*DR5::GUS* (Figure 3e). GUS staining showed that auxin was uniformly distributed in the apical and basal parts of *DR5::GUS* torpedo embryos, but it was asymmetrically distributed in the *GhL1L1*‐deficient or overexpressor torpedo embryos (Figure 3f). To detail the IAA accumulation in embryos, immunolocalization in torpedo embryos with a monoclonal antibody against IAA was performed. The results were similar to the GUS staining results (Figure 3f). In addition, GUS expression was markedly increased in the SAM of the *OE‐GhL1L1*/*DR5::GUS* hybrids compared with the *null*/*DR5::GUS* and *RNAi‐GhL1L1*/*DR5::GUS* hybrids (Figure S5). We conclude that *GhL1L1* affects auxin accumulation and distribution in cotton embryonic tissues.

Polar auxin transport and local auxin biosynthesis determine auxin distribution. To investigate the relationship between *GhL1L1* and auxin distribution, the expression of some auxin biosynthetic genes and polar auxin transport genes were analysed in ECs from *GhL1L1* transgenic and *null* lines. *GhYUC2*,* GhYUC4*,* GhYUC8* and *GhYUC10*, which are key auxin biosynthesis genes, showed irregular changes (Figure S6a–d), indicating that the auxin accumulation was not due to the activation of local auxin biosynthesis. PIN1 and PIN4 mediate auxin efflux and distribution during *Arabidopsis* embryogenesis (Friml *et al*., 2003). The expression level of *GhPIN1* was up‐regulated in ECs from *GhL1L1* overexpression lines but decreased in RNAi lines (Figure 3g), while the transcript of *GhPIN4* showed irregular changes in all lines (Figure S6e). Moreover, the expression of *PHOSPHATASE* 2A (*GhPP2AA2*,* Gh_A11G0044*) was increased in *GhL1L1* overexpression lines (Figure 3h). *GhPP2AA2* is homologous to *AtPP2AA2* (AT3G25800), which functions in the dephosphorylation of PIN auxin efflux carriers (Michniewicz *et al*., 2007; Skottke *et al*., 2011). Thus, we speculated that *GhL1L1* might regulate the expression of *GhPIN1* and *GhPP2AA2* to mediate auxin distribution.

### GhL1L1 binds to the promoter of *GhPP2AA2* to activate its expression

**Figure 4:**
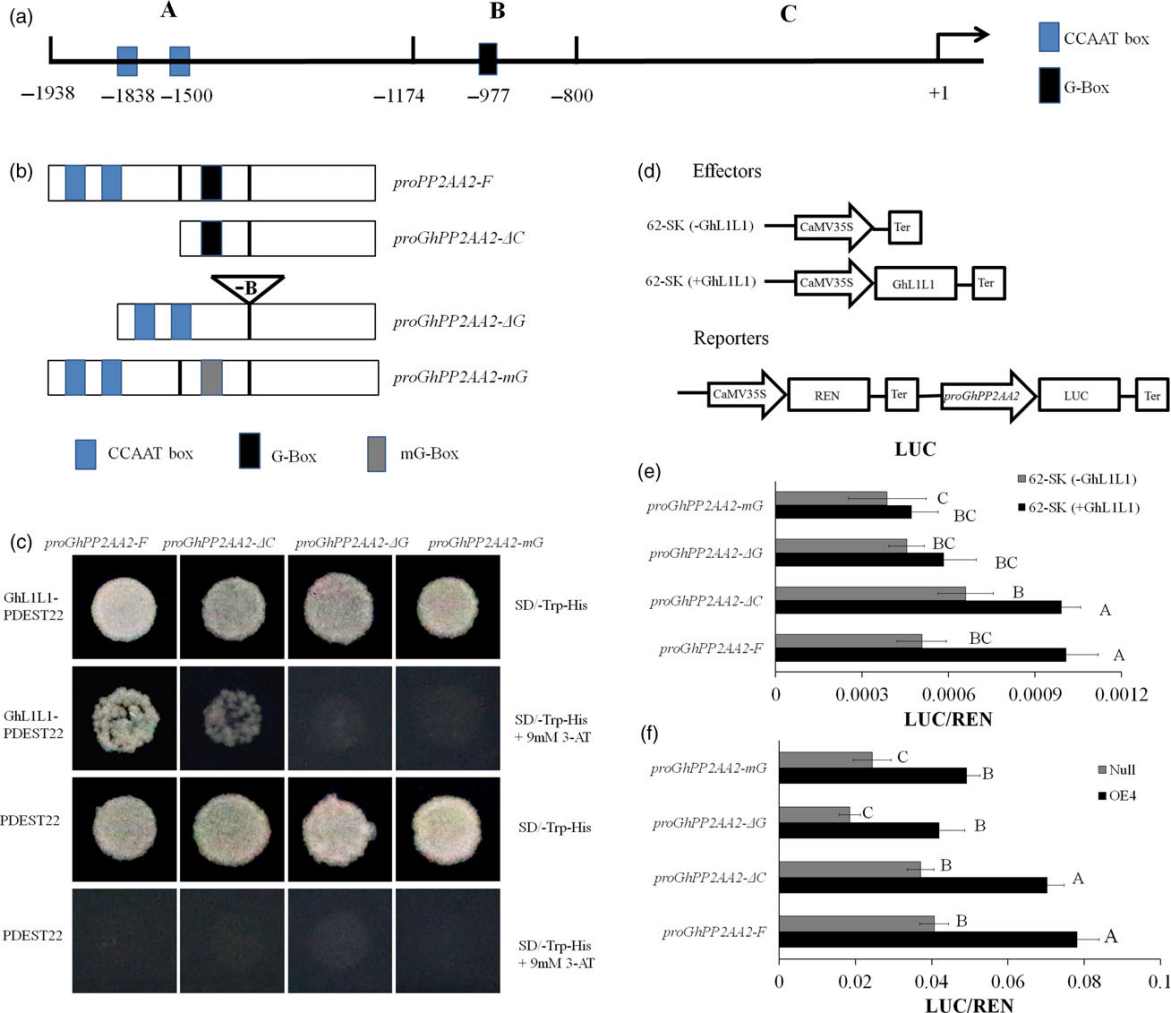
GhL1L1 binds to the G‐box in the promoter to activate the expression of *GhPP2AA2*. (a) Schematic diagram of different regions of the promoter of *GhPP2AA2*, named A, B, C. (b) *proPP2AA2‐F* represents the full length of the promoter, *proGhPP2AA2‐ΔC* represents deletion of the A region*, proGhPP2AA2‐ΔG* represents deletion of the B region and *proGhPP2AA2‐mG* represents mutation of the B region (G‐box, CACGTT mutated to CAAGGT). (c) Transformant yeast colonies visible on the medium (SD‐Trp‐His + 3‐AT) show that GhL1L1 was able to bind to *proPP2AA2‐F and proGhPP2AA2‐ΔC*. No visible yeasts were observed when co‐transforming *GhL1L1* either with *proGhPP2AA2‐ΔG* or *proGhPP2AA2‐mG*. Empty pDEST22 vector was used as a negative control. 3AT, 3‐Amino‐1,2,4‐triazole. (d) Schematic diagram of effectors and reporter. GhL1L1 activates gene expression by binding to the G‐box element in the promoter of *GhPP2AA2* in tobacco leaf protoplasts (e) and OE4 and *null* EC protoplasts (f) *in vivo*. *62‐SK (‐GhL1L1)* served as negative controls. Data are shown as the mean ± SE (*n* = 3). Different capital letters denote significant differences by multiple comparisons using Statistix 8.0 software.

The promoter of *GhPP2AA2* has been cloned and shown to contain two CCAAT‐box motifs and a G‐box motif, which are candidate binding sites for *LEC1‐type* genes (Dorn *et al*., 1987; Li *et al*., 1992; Mendes *et al*., 2013). They were designated A, B and C regions (Figure 4a). Yeast one‐hybrid (Y1H) bait vectors were constructed, named *proGhPP2AA2‐F*,* proGhPP2AA2‐ΔC*,* proGhPP2AA2‐ΔG* and *proGhPP2AA2‐mG* (Figure 4b) and experiments were conducted to study protein‐promoter interactions. Positive results were observed following co‐transformation of *GhL1L1* either with *proGhPP2AA2‐F* or *proGhPP2AA2‐ΔC*, but not with either deficiency or mutation of the G‐box element (Figure 4c).

The dual‐luciferase reporter system was also applied to quantify the interaction between GhL1L1 and *ProGhPP2AA2 in vivo*. The effectors and reporters were transformed into protoplasts from tobacco leaves to exclude the effects of background genes. Simultaneously, the Renilla luciferase (REN) gene driven by the 35S promoter was co‐expressed as an internal control (Figure 4d). Compared with the negative control, GhL1L1 enhanced the activity of the LUC reporter under the control of *proGhPP2AA2‐F* and *proGhPP2AA2‐ΔC* (Figure 4e). *ProGhPP2AA2‐F*,* proGhPP2AA2‐ΔC*,* proGhPP2AA2‐ΔG* and *proGhPP2AA2‐mG* were also used to transform protoplasts from OE4 and *null*. The activation of LUC expression driven by *proGhPP2AA2‐F* and *proGhPP2AA2‐ΔC* was also observed in OE4 (Figure 4f). The results showed that GhL1L1 is able to bind to the G‐box element of the *GhPP2AA2* promoter to activate *GhPP2AA2* expression.

### GhPP2AA2 interacts with GhPIN1 *in vitro* and *in vivo*

**Figure 5:**
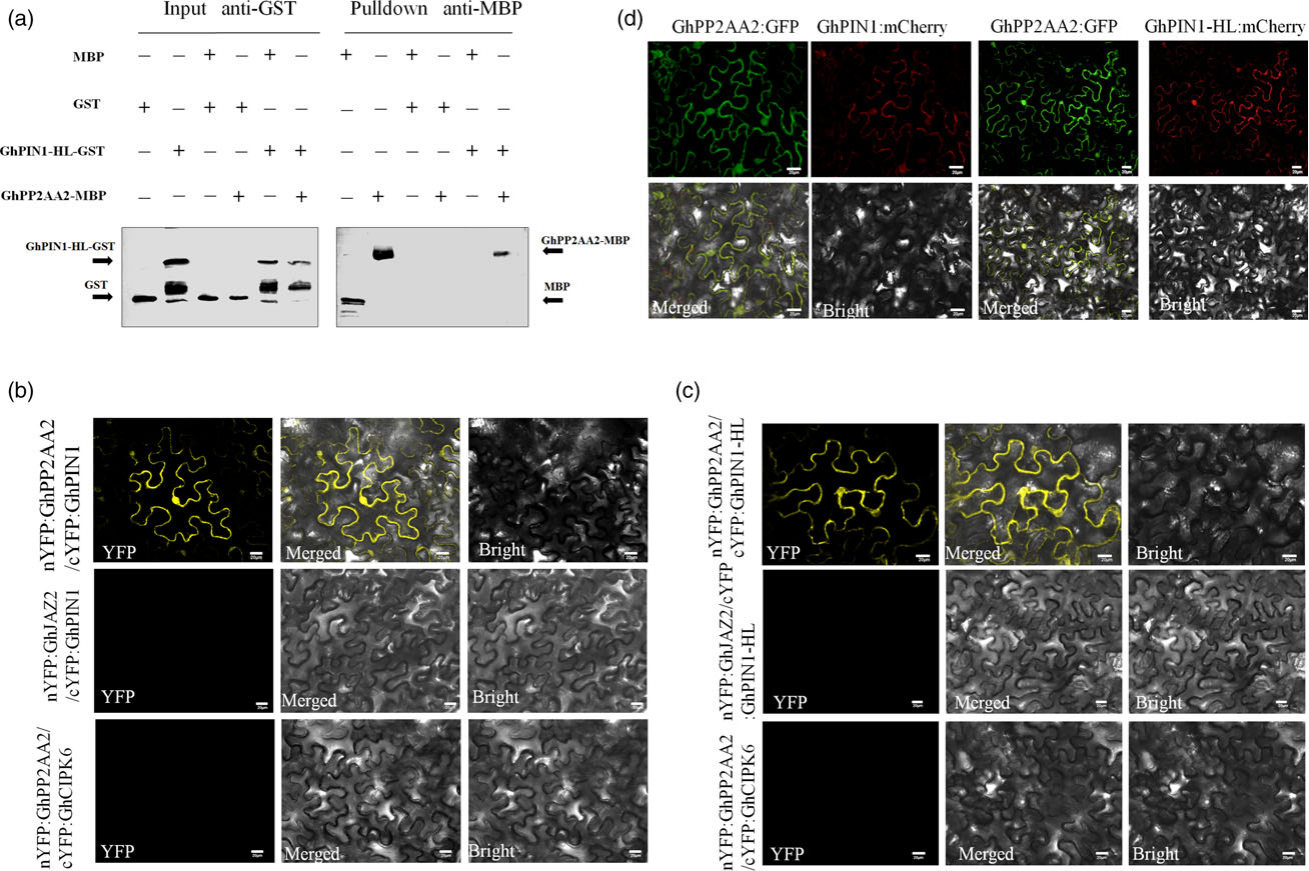
GhPP2AA2 interacts with GhPIN1 protein *in vitro* and *in vivo*. (a) GhPP2AA2 interacts with the GhPIN1 hydrophilic loop (GhPIN1‐HL) in the pull‐down assay *in vitro*. GST and GST‐GhPIN1‐HL proteins were used to pull down interacting proteins. MBP or MBP‐GhPP2AA2 proteins were detected by Western blotting with anti‐MBP antibodies and anti‐GST as input. BiFC assays revealed the interaction of GhPP2AA1 with GhPIN1 (b) and GhPIN1‐HL (c) *in vivo*. Yellow fluorescence (YFP) indicated a positive interaction. nYFP:GhJAZ2 and cYFP:GhCIPK6 fusion proteins served as negative controls. (d) GhPP2AA2 and GhPIN1 (GhPIN1‐HL) colocalized as indicated by GFP and mCherry. Scale bars = 20 μm in b, c and d.

PP2A phosphatase was identified as an important regulator of PIN activity and auxin distribution (Michniewicz *et al*., 2007). To understand the relationship between GhPP2AA2 and GhPIN1, both *in vitro* and *in vivo* experiments were performed. To obtain GhPIN1 protein, recombinant protein GhPIN1‐HL‐GST was produced by removing the transmembrane domain from the hydrophilic loop of GhPIN1 (GhPIN1‐HL). GhPIN1‐HL was fused to glutathione S‐transferase (GST) and GhPP2AA2 was fused to maltose‐binding protein (MBP). *In vitro* pull‐down assays showed that recombinant GhPIN1 and GhPP2AA2 interacted with each other (Figure 5a). These interactions were further confirmed by bimolecular fluorescence complementation (BiFC) assays *in vivo*. Strong YFP fluorescence signals indicated that GhPP2AA2 interacted with GhPIN1‐HL (Figure 5b,c). Moreover, tobacco epidermal cells were transformed with the FRET 2in1 vectors, GhPP2AA2 fused to GFP and GhPIN1 or GhPIN1‐HL fused to mCherry, which showed that GhPP2AA2 and GhPIN1 colocalized at the cell membrane (Figure 5d). These results indicated that GhPIN1 directly interacts with GhPP2AA2.

### Disrupted trafficking of GhPIN1 inhibits auxin polar transport and accelerates cell fate specification during embryo formation

**Figure 6:**
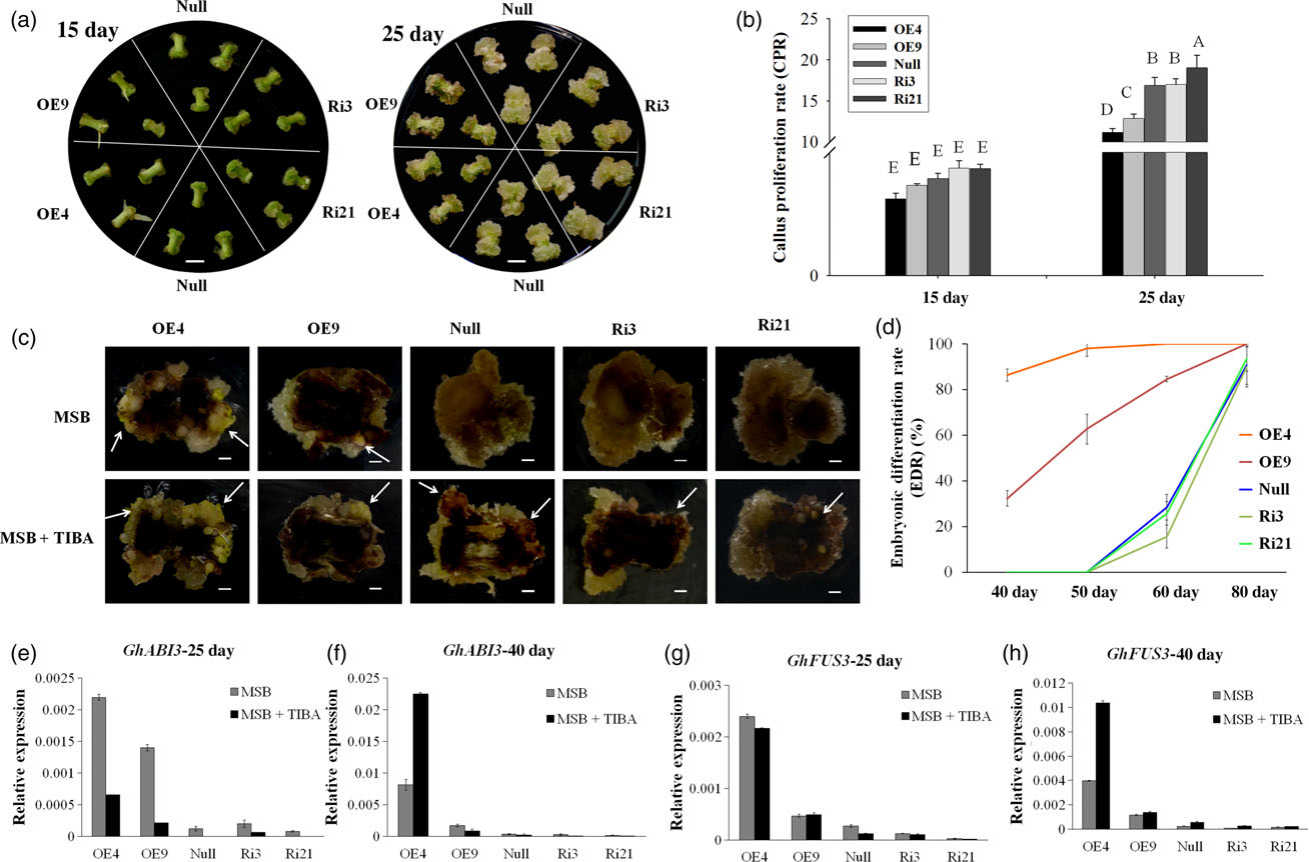
TIBA treatment affects callus proliferation and auxin distribution during cotton SE. (a) The phenotypes of different *GhL1L1* transgenic and *null* lines at 15 and 25 days postinduction treated with TIBA, scale bars = 5 mm. (b) The CPR of different transgenic and *null* lines at 15 and 25 days postinduction treated with TIBA. Different capital letters denote significant differences by multiple comparisons using Statistix 8.0 software. (c) ECs or embryos were observed from the transgenic and *null* lines (white arrows) at 50 days postinduction treated with TIBA, scale bars = 2 mm. (d) The embryonic differentiation rate (EDR) of different transgenic and *null* lines at 40, 50, 60 and 80 days postinduction treated by TIBA. qRT–PCR analysis of *GhABI3* (e, f) and *GhFUS3* (g, h) in *GhL1L1* transgenic and *null* explants induced on MSB medium or supplemented with TIBA at 25, 40 days postinduction. The data (in b, d, e, f, g and h) are shown as the mean ± SE (*n* = 3).

To confirm whether disrupted PIN1 protein affects cotton cell fate, a synthetic inhibitor of PIN1, TIBA, was added to MSB medium to culture *GhL1L1* transgenic and *null* explants. CPR was measured at 15 and 25 days postinduction. As shown previously, CPR of *OE4* and *OE9* was less than *null*, while CPR of *Ri21* increased on MSB medium without TIBA (Figure 2c). However, callus proliferation was suppressed by TIBA in all the lines, especially in RNAi lines, and the difference in CPR among different lines could not be clearly observed at 15 days postinduction. However, callus growth between the apical and basal parts of the explants in RNAi lines was poorly differentiated (Figure 6a,b). TIBA treatment accelerated the presence of ECs in all lines, but especially in RNAi lines, where they were observed at least 10 days earlier than in other lines. EDR was increased to 86.4% in OE4 and 32.3% in OE9 at 40 days post‐induction, and it was increased to at least 90% in *null* and RNAi lines at approximately 80 days postinduction (Figures 2e and 6c,d). Moreover, the expression level of *GhABI3* and *GhFUS3* increased to a high level at 40 days postinduction in overexpression line OE4, in accordance with the phenotype (Figure 6e–h).

TIBA treatments were also applied to *DR5::GUS* transgenic seedlings during cotton SE. GUS staining was uniformly distributed at both ends of the explants before induction, while it was accumulated to the morphological basal parts of explants at 1, 3, 7, 15 and 40 days postinduction without TIBA treatment (Figure S7a), demonstrating that the auxin distribution polarized to the morphological basal region of the explants. When TIBA was added to the medium, the polar distribution of auxin was disturbed, with most of the GUS staining accumulating in the morphological basal parts of the explants, with little diffusion to the morphological apical part (Figure S7b), similar to the overexpression explants of *GhL1L1* (Figure 2a) and the *OE4/DR5::GUS* explants induced on MSB medium (Figure S7c). These results suggest that *GhL1L1* overexpression may disturb auxin polar distribution, to accelerate cell fate specification during embryo formation.

## Discussion

### GhL1L1 displays a conserved B domain and specific expression during embryogenesis

*LEC1* encodes a CCAAT‐binding transcription factor of the HAP3 subunit. LEC1 and L1L are LEC1‐type subunits with a conserved B domain (Kwong *et al*., 2003). The B domain of LEC1 and L1L is necessary for its activity in embryogenesis (Lee *et al*., 2003). We demonstrate that GhL1L1 and other LEC1‐type proteins are highly conserved in the B domain (Figure S1). LEC1 is required during seed maturation, and embryos of the *lec1* mutant are intolerant to seed desiccation (Lotan *et al*., 1998). The expression pattern of *L1L* is similar to that of *LEC1* (Kwong *et al*., 2003). Additionally, genes such as *LEC2*,* FUS3* and *ABI3*, closely related B3 domain transcription factors, have been reported to play major roles in embryo maturation (Braybrook *et al*., 2006; Stone *et al*., 2001). We found that the expression of *GhL1L1* was only detected in seeds (Figure S2b) or embryonic cells (Figure 1a). These results show that GhL1L1 is highly conserved and specifically expressed in cotton. Additionally, GhL1L1 is an important player in embryogenesis.

### Somatic embryogenesis accompanied by complex auxin dynamics

Somatic embryogenesis is a process whereby somatic cells regenerate into embryos, then to new plants by *in vitro* culture without fertilization. Callus induction is fundamental to cotton SE, which is the foundation of producing transgenic cotton via *Agrobacterium*‐mediated transformation. The molecular mechanisms underlying callus induction are complex. The molecular mechanisms underlying callus induction have been documented, including auxin induction, cytokinin induction, wound induction and formation via the reacquisition of embryonic or meristematic fates (Ikeuchi *et al*., 2013). Callus formation is associated with a variety of hormones. An intermediate ratio of auxin and cytokinin can increase callus induction and proliferation (Skoog and Miller, 1957). Brassinosteroid, abscisic acid or ethylene also can induce callus formation in some plants (Goren *et al*., 1979; Hu *et al*., 2000; Wang *et al*., 2018). TIBA inhibits auxin distribution and PIN1 localization, which are important for embryogenesis (Forestan *et al*., 2010; Geldner *et al*., 2001). Auxin levels change dynamically during cotton SE (Yang *et al*., 2012). The strictly polar auxin distribution can be observed in the explants of *DR5::GUS* induced on MSB medium (Figure S7a). However, polar auxin transport was disrupted after treatment with TIBA (Figure S7b), the proliferation of NECs was repressed by TIBA (Figure 6c), while the embryonic cell formation was advanced by TIBA treatment (Figure 6c,d). We propose that cell fate specification accompanied by complex auxin dynamics, encompassing not only auxin levels but also auxin distribution.

### GhL1L1 affects auxin polar distribution

GhL1L1 is an important regulator for cell fate specification. Overexpression of *GhL1L1* accelerated embryonic cell formation and restricted callus proliferation, with altered auxin distribution. By contrast, *GhL1L1*‐deficient explants dedifferentiated vigorously but showed retarded embryonic cell formation (Figure 2). A new idea presented here is that *GhL1L1* regulates auxin distribution during cell fate specification. In this study, the concentration of free IAA increased in ECs and SAM after overexpressing *GhL1L1* (Figures 3 and S5). Additionally, the GUS staining increased in *GhL1L1* overexpression lines but decreased in *GhL1L1* RNAi lines (Figure 3), paralleled with the polar growth of explants in transgenic lines. This indicates that *GhL1L1* participates in cell fate specification by regulating auxin distribution. Moreover, the postinduction phenotype of explants from *GhL1L1* overexpression lines was similar to the postinduction phenotype of the wild type treated with TIBA. Furthermore, the polarized growth of callus on the both ends of explants was decreased. The calluses of *GhL1L1* overexpression lines were more dramatically suppressed (Figure 6a,b).

Gradients of auxin are mediated by its efflux via asymmetrically localized PIN proteins (Benková *et al*., 2003; Paponov *et al*., 2005). Polar auxin transport and correct apical–basal axis formation of the embryo require PIN1, PIN4 and PIN7 (Friml *et al*., 2003; Guenot *et al*., 2012). The expression of *GhPIN1* was altered in ECs of the *GhL1L1* overexpression lines (Figure 3g), and auxin accumulated. Hence, we suppose that auxin efflux might be decreased because of the effect on GhPIN1 activity, resulting in an increased auxin concentration. PIN polarity is related to the phosphorylation status of PIN proteins. The absence of PP2A, in particular PP2AA1, PP2AA2 or PP2AA3, increases PIN1 phosphorylation in embryos. PP2A regulates the dephosphorylation of PIN proteins (Friml *et al*., 2004; Michniewicz *et al*., 2007). Our data confirm that GhL1L1 positively regulates GhPP2AA2 to affect the activity of GhPIN1 (Figure 4). Thus, overexpression of *GhL1L1* or treatment with TIBA affected the auxin polar transport by affecting the activity of GhPIN1.

## Conclusion

**Figure 7:**
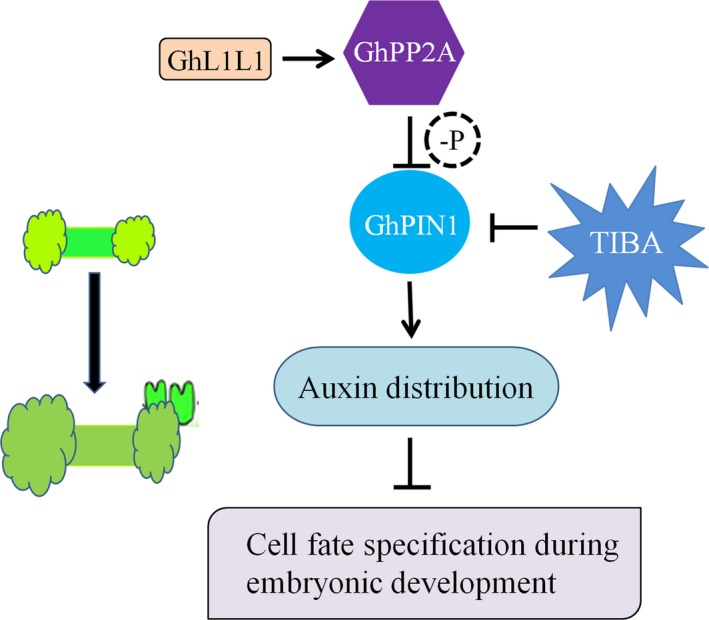
Schematic showing the role of *GhL1L1* during cotton SE. GhL1L1 activates the expression of *GhPP2AA2*, which might dephosphorylate GhPIN1, which was also affected by TIBA treatment, to affect the auxin distribution, and then affects cell fate specification during embryonic development.

Based on an integration of the relationships between different morphological and biochemical changes in cotton, GhL1L1 repressed the initial cell dedifferentiation and callus proliferation, but it played a positive role in embryonic cell formation. GhL1L1 activated the expression of *GhPP2AA2* by binding to the cis‐element G‐box in the promoter of *GhPP2AA2* to interact with GhPIN1 protein and affect the activity of GhPIN1, which was also affected by TIBA treatment (Figure 7).

## Experimental procedures

### GhL1L1 sequencing analysis, vectors construction and transformation

The full‐length *GhL1L1* was obtained from cDNA amplification of cotton ECs. The amino acid sequence alignments and phylogenetic relationship were analysed with the ClustalX and MEGA6 software respectively. The coding sequence was inserted into the vector PK2GW7 to construct the vector *35S::GhL1L1* by Gateway Technology (Invitrogen, Carlsbad, CA) for overexpression. The 3′ untranslated region and coding region were cloned into the RNAi vector pHellsgate4 by recombination reaction. The overexpression and RNAi vectors were transformed into *G. hirsutum* ‘YZ1’ plants *via Agrobacterium tumefaciens* (EHA105) as described previously (Jin *et al*., 2005, 2006). All primers used in the vectors construction are listed in Table S1.

### Plant materials, callus induction and TIBA treatment

Cotton materials, YZ1 [cotton (*G. hirsutum*)], Jin668 [cotton (*G. hirsutum*)], Simian3 [cotton (*G. hirsutum*)], H7124 [cotton (*G. barbadense*)], 3–79 [cotton (*G. barbadense*)], *DR5::GUS* transgenic plants, *GhL1L1* transgenic plants and F_1_ hybrids of *DR5::GUS* transgenic plants with *GhL1L1* transgenic plants were used in this study. The *DR5::GUS* and *GhL1L1* transgenic plants were in the YZ1 background. The callus induction procedure was performed as follows. Hypocotyls of etiolated seedlings sampled at 0 h or cut into 5–7‐mm sections as explants were cultured on MSB medium at different time points or subcultured for EC and somatic embryos as described previously (Yang *et al*., 2012). The MSB medium was supplemented with 5 μm TIBA (Sigma, St. Louis, MO) to monitor the disruption of the auxin distribution.

### Southern and northern blotting, RT–PCR and qRT–PCR

To determine the copy number of T‐DNA inserted in transgenic cotton, Southern blotting was performed as previously described (Li *et al*., 2010). Genomic DNA was extracted from leaves of transgenic cotton using the Plant Genomic DNA Kit (Tiangen Biotech, Beijing, China). Approximately, 15 μg of DNA was hybridized with the probe of an NPTII fragment using a DIG‐High Prime DNA Labeling and Detection Starter Kit II (Roche, Mannheim, Germany).

To determine the expression level of *GhL1L1* in wild‐type and transgenic plants, total RNA was isolated from ECs and leaves using a PureLink RNA Mini Kit (Invitrogen). Northern blotting was performed as previously described (Tu *et al*., 2007). Approximately, 15 μg of RNA was hybridized with a *GhL1L1* probe fragment labelling with the DIG‐High Prime DNA Labeling and Detection Starter Kit II. RT‐PCR and qRT‐PCR were performed as previously described (Hao *et al*., 2012). The expression level of *GhUBQ7* (DQ116441) was used as the internal control (Tu *et al*., 2007). The primers used for Southern and northern blotting, RT–PCR and qRT–PCR are listed in Table S1.

### Callus proliferation rate and EDR calculation

The hypocotyl of *GhL1L1* transgenic plants and *DR5::GUS* were cut into approximately 7 mm pieces and induced on MSB medium or supplemented with 5 μm TIBA in the culture room. The CPR was calculated as the fold change in weight gained of explants at 15 and 25 days postinduction as described (Wang *et al*., 2018). The EDR (the explants with ECs or embryos/the total explants) was calculated by the rate of ECs or embryos occurred on the explants at 40, 50, 60 and 80 days postinduction on MSB medium or supplemented with 5 μm TIBA.

The CPR and EDR experiments were conducted with three biological replicates, and each replicate represented at least four culture dishes with at least 10 explants each dish.

### GUS assay and histochemical analysis

The hypocotyl of *GhL1L1* transgenic plants induced on MSB medium or supplemented with 5 μm TIBA were photographed using a Nikon D40 camera (Nikon corporation, Tokyo, Japan) at 15 and 25 days postinduction. Calluses were removed and stained with propidium iodide to visualize cellular structure at 40 days postinduction, and the features were photographed under a microscope (Zeiss, Oberkochen, Germany). The hypocotyls of five F_1_ hybrids (*OE4/DR5::GUS, OE9/DR5::GUS, Ri3/DR5::GUS, Ri21/DR5::GUS, null/DR5::GUS*) were induced on MSB medium. Histochemical localization and quantitative analyses of GUS activity was performed as described previously (Cai *et al*., 2008; Deng *et al*., 2012). The features of GUS staining were observed and photographed under a microscope (Leica, Wetzlar, Germany). To evaluate the microstructure of torpedo embryos after GUS staining and the structure of hypocotyls, samples were fixed in 50% FAA and cut into 8‐μm‐thick sections as in a previous study (Yang *et al*., 2012). GUS‐stained sections were directly observed after being deparaffinized with xylene. To observe the structure of the hypocotyl, the deparaffinized hypocotyl sections were stained with 1% toluidine blue. The photographs were obtained under a microscope (Zeiss). To observe the structure of transgenic EC cells, transmission electron microscope analysis was performed as previously described (Sun *et al*., 2014). The experiments were conducted with three biological replicates.

### Endogenous IAA extraction, quantification and immunofluorescence localization

To estimate the concentration of endogenous IAA, samples were immediately ground in liquid nitrogen and extracted in 1 mL 80% cold methanol, which contains 10 ng/mL ^2^H_5_‐IAA (Olchemlm, Olomouc, Czech Republic), as an internal standard. Further extraction and quantitative analyses of IAA were performed as described previously (Liu *et al*., 2012). The experiments were conducted with three biological replicates.

Samples of torpedo embryos were fixed in 50% FAA as described previously (Hou and Huang, 2005). Sections were incubated with anti‐rabbit Dylight 488 secondary antibody (Thermo Scientific, Waltham, MA) for immunofluorescence. Fluorescence was assayed using a confocal laser‐scanning microscope (Olympus, Tokyo, Japan).

### Yeast one‐hybrid assay

To characterize the interaction between GhL1L1 and the promoter of *GhPP2AA2* in yeast, the promoter sequence of *GhPP2AA2* (−1 to −1939) was amplified by PCR using YZ1 genomic DNA and cloned into the pHis‐1 bait vector to generate pHis‐1‐*proGhPP2AA2*. It was then divided to three regions: −1174 to −1953‐bp region containing two CCAAT boxes (at approximately −1838, −1500 bp) named A, −800 to −1173‐bp region containing a G‐box motif (at approximately −977 bp) named B and −1 to −799‐bp region containing basic promoter elements without the CCAAT‐box motif and G‐box named C. Three vectors of the promoters were constructed in the pHis‐1 bait vector, with *proGhPP2AA2‐ΔC* representing deletion of the A region*, proGhPP2AA2‐ΔG* representing deletion of the B region and *ProGhPP2AA2‐mG* representing mutation of the B region (G‐box, CACGTT mutated to CAAGGT). The Y1H assay was performed as previously described (Min *et al*., 2015). The primers used in the Y1H assay are listed in Table S1.

### Dual‐luciferase reporter assay system

The transient dual‐luciferase reporter assays were performed as described previously (Hellens *et al*., 2005). The full‐length and three‐variant promoters (*proGhPP2AA‐F, proGhPP2AA2‐ΔC, proGhPP2AA2‐ΔG* and *proGhPP2AA2‐mG*) were cloned into pGreenII 0800‐LUC at the PstI and BamHI sites. Moreover, *GhL1L1* was cloned into vector *62‐SK* to obtain *62‐SK* (*+GhL1L1*). These plasmids and *62‐SK* (*+GhL1L1*) or negative *62‐SK* (*−GhL1L1*) were co‐transformed into protoplasts from tobacco leaves. These plasmids were also transformed into protoplasts from ECs of OE4 and *null* respectively. Firefly luciferase and *Renilla* spp. luciferase activities were then quantified using the dual‐luciferase assay reagents (Promega, Madison, WI) with a Multimode Plate Reader (PerkinElmer). The primers used in the LUC assays are listed in Table S1.

### *In vitro* pull‐down assay

PIN1 is a membrane protein and is difficult to express in prokaryotes (Michniewicz *et al*., 2007). CDS deletion of the transmembrane domain of GhPIN1 (*GhPIN1‐HL*) was cloned and constructed into pGEX‐4T‐1 to obtain GST‐GhPIN1‐HL recombinant proteins. For MBP‐GhPP2AA2 recombinant proteins, the CDS of *GhPP2AA2* was cloned into pMAL‐c4x. The GST fusion proteins and MBP fusion proteins were purified using glutathione beads (Promega) and amylose resin (NEB, Ipswich, MA). The pull‐down assay was performed as described previously (Yang *et al*., 2017). The primers used in the pull‐down assay are listed in Table S1.

### Bimolecular fluorescence complementation assays and colocalization

Bimolecular fluorescence complementation assays were performed as described previously (Grefen and Blatt, 2012). The sequences of *GhPP2AA2* and *GhPIN1* (*or GhPIN1‐HL*) were constructed in *pDONR221* via recombination reactions. The pBIFCt‐2in1‐NN vectors were constructed via attL and attR site (LR) recombination (Invitrogen) for BiFC. nYFP:GhJAZ2 (nuclear‐localized protein) and cYFP:GhCIPK6 (membrane‐localized protein) fusion proteins were used as negative control. The pFRETgc‐2in1‐NN vector was constructed by LR recombination for colocalization (Hecker *et al*., 2015). All the vectors were transformed into *Agrobacterium tumefaciens* GV3101 and used to infect tobacco epidermal cells. The YFP, GFP and mCherry fluorescence were assayed using a confocal laser‐scanning microscope (Olympus). The primers used in the BiFC assays and colocalization were listed in Table S1.

### Statistical analysis

All experiments were conducted with at least three biological replicates, and the values are displayed as the mean ± SD. Statistical significance was determined using Student's *t‐*test, *P < 0.05; **P < 0.01 were considered statistically significant, and multiple comparisons were performed using Statistix 8.0 software (Analytical Software, Tallahassee, FL ).

## Conflicts of interest

The authors declare no conflict of interest.

## Supporting information


**Figure S1** Alignment analysis of LEC1‐type subunit.
**Figure S**2 NF‐YB subfamily in cotton.
**Figure S3** Expression analysis by qRT‐PCR.
**Figure S4** Southern blotting of transgenic cotton plants.
**Figure S5** GUS staining of the shoot apical meristem (SAM).
**Figure S6** qRT‐PCR analysis of the genes expression.
**Figure S7** GUS staining of *DR5::GUS* explants.
**Table S1** The primers used in this study.
