# Supplementary material for: GhL1L1 affects cell fate specification by regulating GhPIN1‐mediated auxin distribution
Source: Plant Biotechnol J. 2018 May 31;17(1):63–74. doi: 10.1111/pbi.12947 (PMC6330550; doi:10.1111/pbi.12947)
Supplement: Supplementary file 1 — Figure S1 Alignment analysis of LEC1‐type subunit. Figure S2 NF‐YB subfamily in cotton. Figure S3 Expression analysis by qRT‐PCR. Figure S4 Southern blotting of transgenic cotton plants. Figure S5 GUS staining of the shoot apical meristem (SAM). Figure S6 qRT‐PCR analysis of the genes expression. Figure S7 GUS staining of DR5::GUS explants. Table S1 The primers used in this study. [file PBI-17-63-s001.doc]

**Supporting Figures**

**
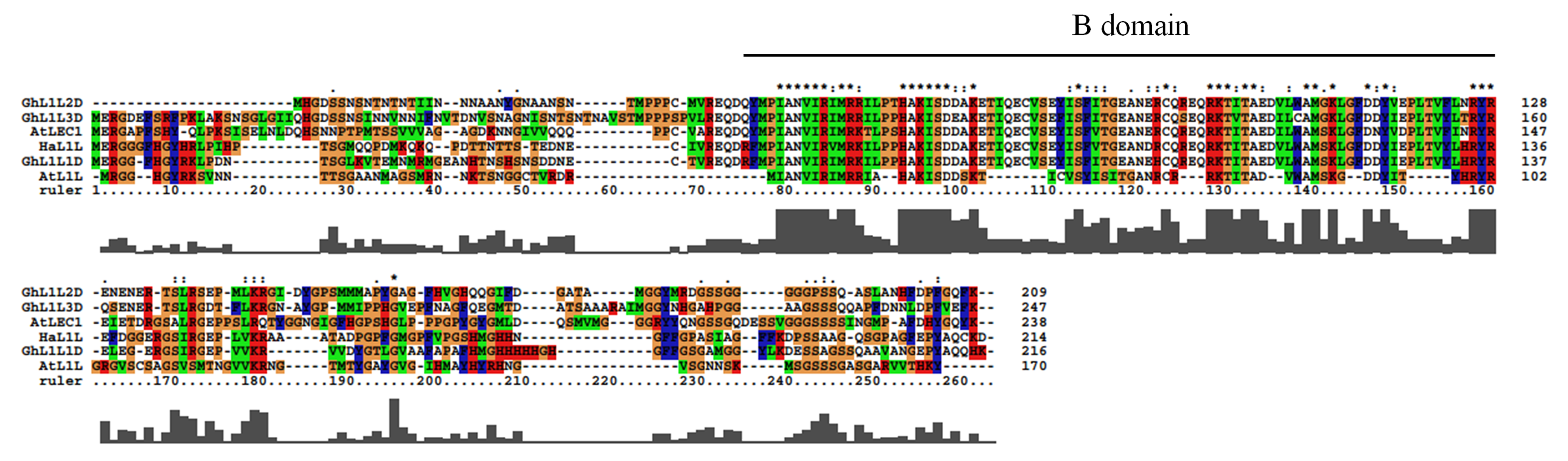
**

Figure S1. **Alignment analysis of LEC1-type subunit.** The LEC1-type amino acid sequences of the different species. The horizontal line indicates the B domain.


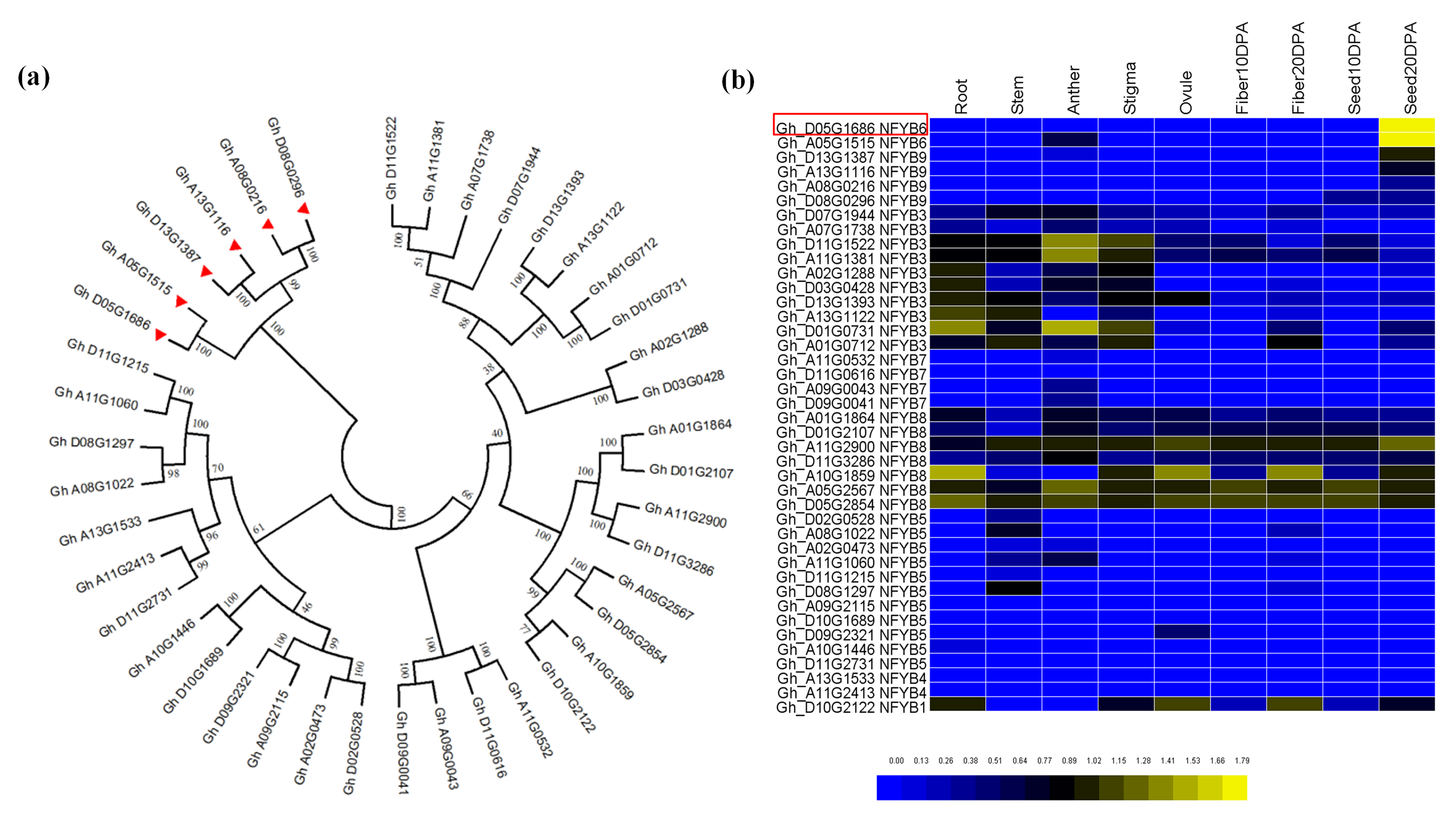


Figure S2. NF-YB subfamily in cotton. (a) The phylogenetic relationship between LEC1-type proteins (red triangle) and non-LEC1-type proteins in NF-YB subfamily proteins from *Gossypium hirsutum* (TM-1). (b). Heat map of the expression of 41 NF-YB genes in 9 tissues, including the root, stem, anther, stigma, ovule, fibre 10 DPA, fibre 20 DPA, seed 10 DPA, seed 20 DPA.


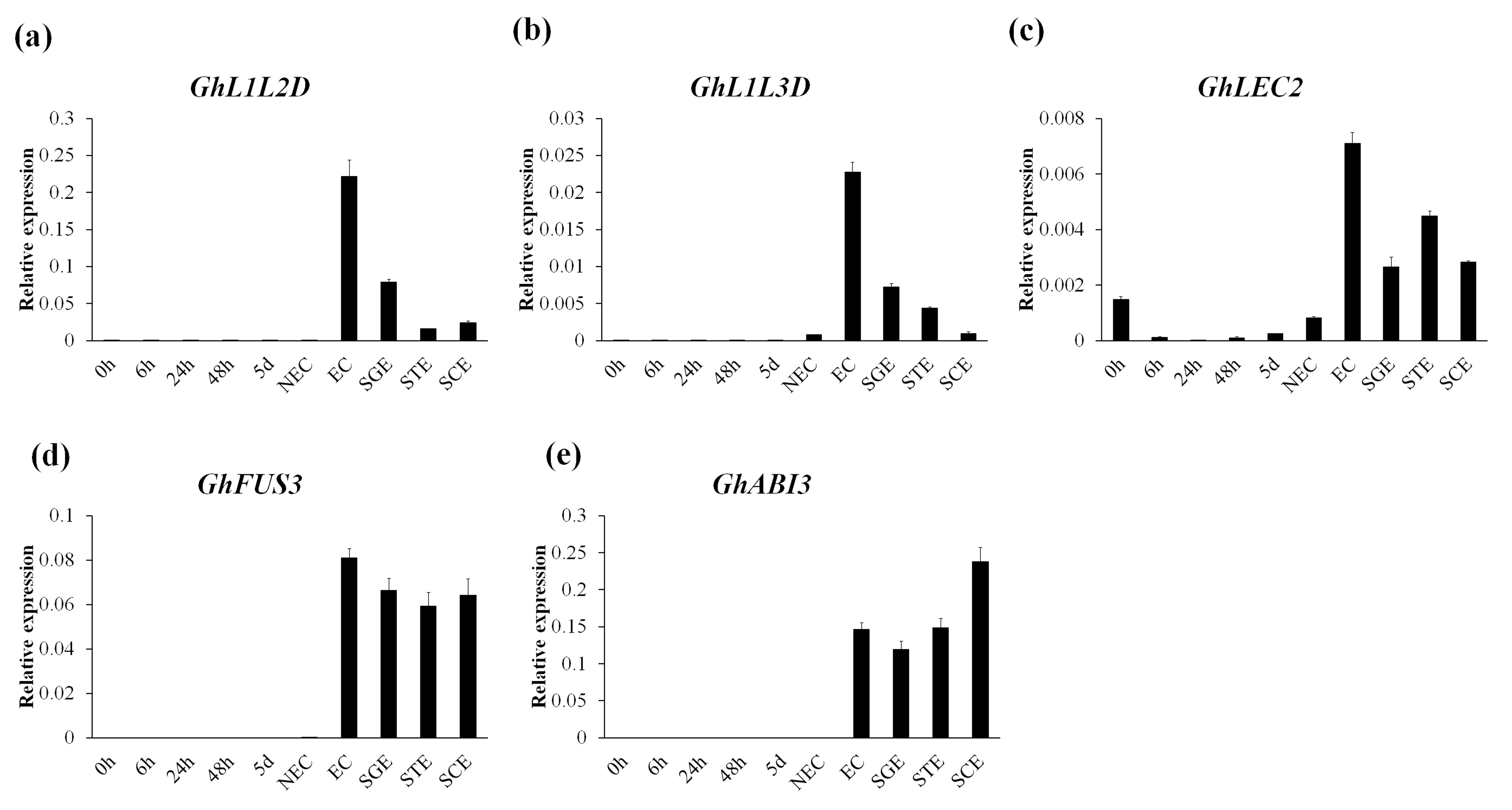


Figure S3. **Expression analysis by qRT-PCR.** (a and b) The expression of *GhL1L1* homologous genes during cotton SE by qRT-PCR analysis. (c-e) The expression of the B3 group of transcription factors during cotton SE by qRT-PCR analysis.


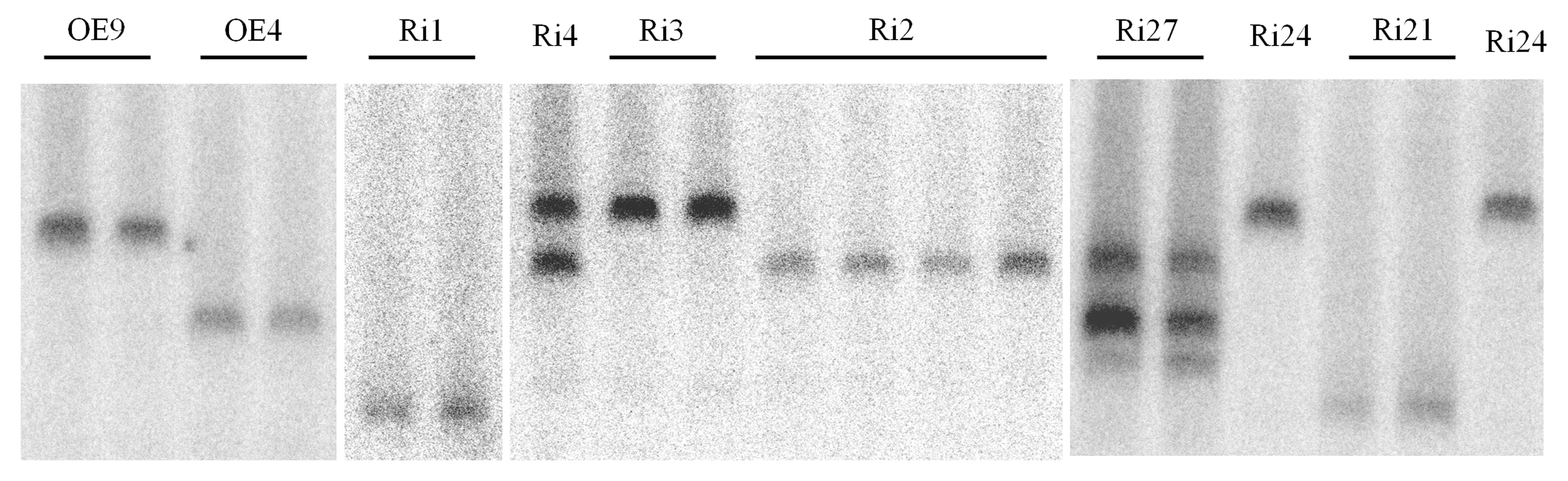


Figure S4. **Southern blotting of transgenic cotton plants.** OE4 and OE9 represent the overexpression lines, Ri1, Ri2, Ri3 and Ri4 represent RNA interference of 3’ untranslated region lines, Ri21, Ri24 and Ri27 represent RNA interference of coding region lines.


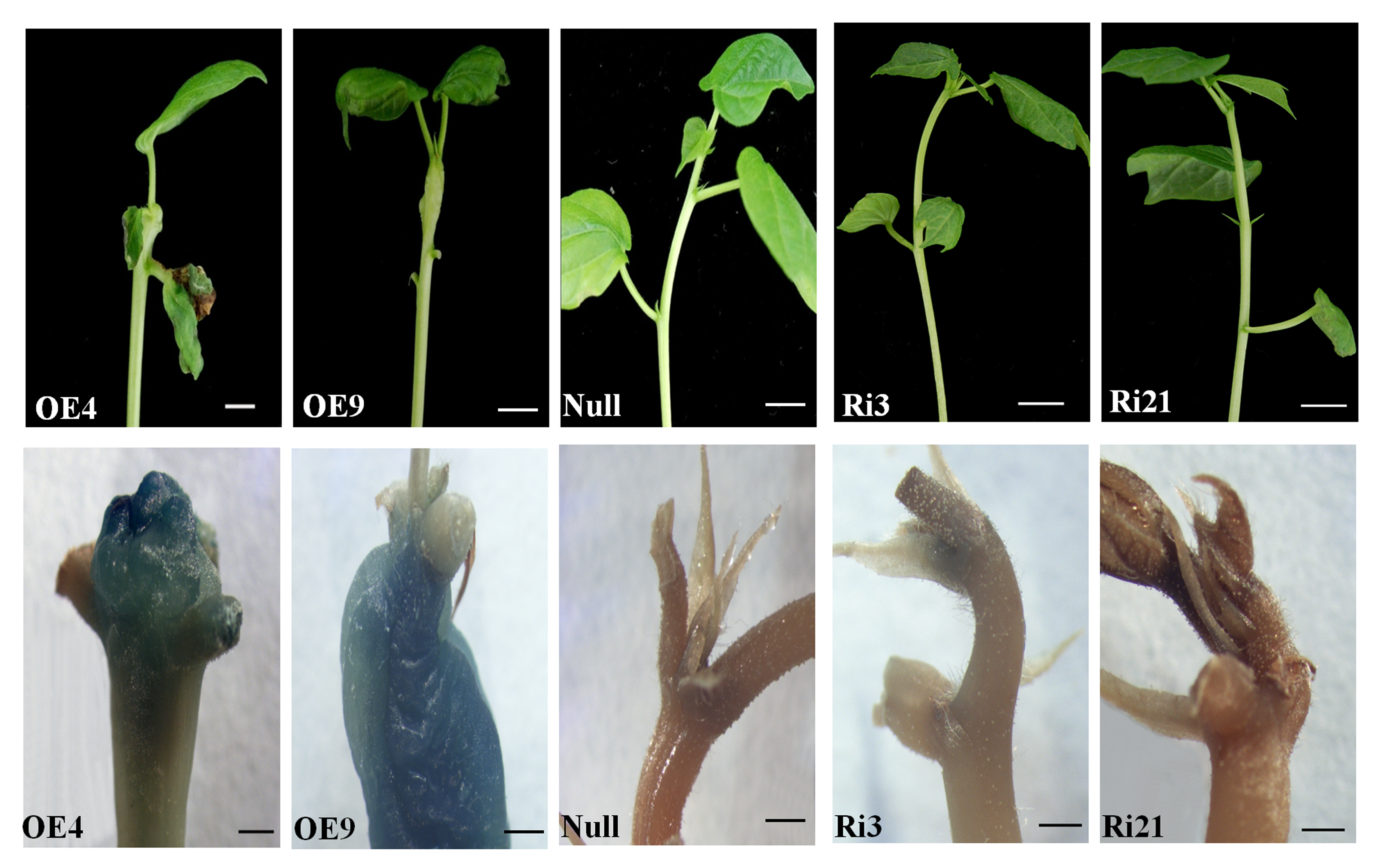


Figure S5. GUS staining of the shoot apical meristem (SAM).GUS staining of the SAM in the five F_1_ hybrids (OE4/DR5::GUS, OE9/DR5::GUS, Ri3/DR5::GUS, Ri21/DR5::GUS, null/DR5::GUS), upper scale bars = 1 cm, below scale bars = 2 mm.


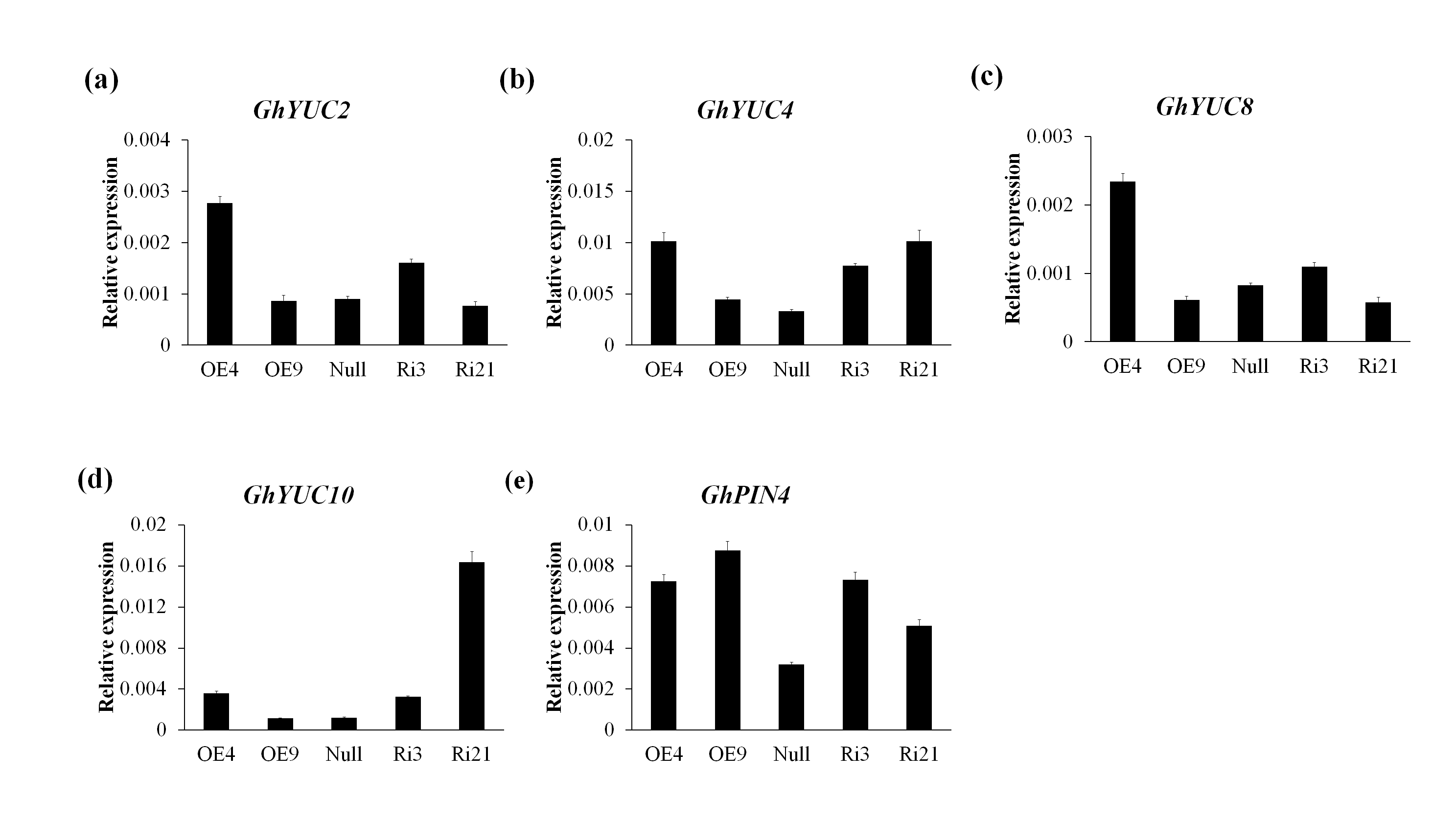


Figure S6. qRT-PCR analysis of the genes expression. (a-d) The expression level of auxin synthesis genes *GhYUCs* in ECs of *GhL1L1* transgenic (OE4, OE9, Ri3 and Ri21) and *null* lines by qRT-PCR analysis. (e) The expression level of *GhPIN4* in ECs of *GhL1L1* transgenic and *null* lines by qRT-PCR analysis.


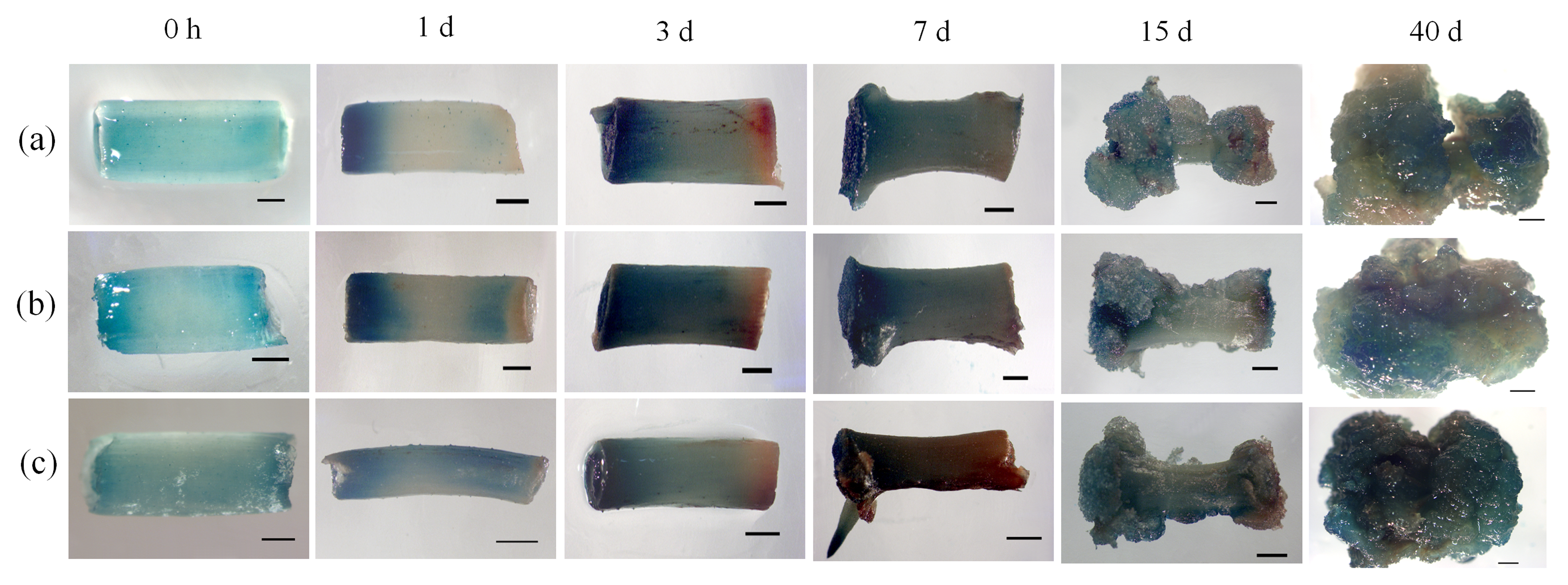


Figure S7. GUS staining of *DR5::GUS* explants. *DR5::GUS* hypocotyls were cultured on MSB medium (a) or supplemented with TIBA (b) and *OE4/DR5::GUS* hypocotyls on MSB medium (c) at 0, 1, 3, 7, 15 and 40 days post-induction.

**Supporting Table**

**Table S1. The primers used in this study.**

| **Primer name** **Sequences (5'-3')** | | | **Destination** |
| --- | --- | --- | --- |
| ***GhL1L1*-F**  ***GhL1L* -R**  ***GhL1L1*-OE-F**  ***GhL1L1-*OE*-*R**  ***GhL1L1*-UTR-RNAi-F**  ***GhL1L1*-UTR-RNAi-R**  ***GhL1L1*-ORF-RNAi--F**  ***GhL1L1*-ORF-RNAi--R** | GAGAGGGGGATCCAGCTACAGGAG  GAAGCAGAAGTCAATGCAGGTC  GGGGACAAGTTTGTACAAAAAAGCAGGCTACAAGGAGAGACAGATGGAACGTGGA  GGGGACCACTTTGTACAAGAAAGCTGGGTCGAGACCGCGGCCTCATTTATG  GGGGACAAGTTTGTACAAAAAAGCAGGCTCCTGGTTGATTATGGAACTTTGGG  GGGGACCACTTTGTACAAGAAAGCTGGGTCGAAGCAGAAGTCAATGCAGGTC  GGGGACAAGTTTGTACAAAAAAGCAGGCTACTGCGCAAGATCCTTCCCCCAC  GGGGACCACTTTGTACAAGAAAGCTGGGTCGAGACCGCGGCCTCATTTATG |  | *GhL1L1* gene cloning  *GhL1L1* Over-expression or Y1H  *GhL1L1* 3'-UTR region RNAi  *GhL1L1* coding region RNAi |
| **GhL1L1-Northern-F**  **GhL1L1-Northern-R**  **NPT-Ⅱ-F**  **NPT-Ⅱ-R**  ***GhL1L1*-RT-F**  ***GhL1L1*-RT-R**  ***GhL1L2*-F**  ***GhL1L2*-R**  ***GhL1L3*-F**  ***GhL1L3*-R**  ***GhLEC2*-F**  ***GhLEC2*-R**  ***GhABI3*-F**  ***GhABI3*-R**  ***GhARF19*-F**  ***GhARF19*-R**  ***GhIAA33-*F**  ***GhIAA33-*R**  ***GhPIN4*-F**  ***GhPIN4-*R**  ***GhPIN1*-F**  ***GhPIN1*-R**  ***GhYUC2*-F**  ***GhYUC2*-R**  ***GhYUC4*-F**  ***GhYUC4*-R**  ***GhYUC8*-F**  ***GhYUC8*-R**  ***GhYUC10*-F**  ***GhYUC10*-R**  ***UBQ7*-F**  ***UBQ7*-R**  ***proGhPP2AA2*-F**  ***proGhPP2AA2*-R**  ***proGhPP2AA2-ΔC*-F**  ***proGhPP2AA2-ΔC-*R**  ***proGhPP2AA2-ΔG1*-F**  ***proGhPP2AA2-ΔG1*-R**  ***proGhPP2AA2-ΔG2*-F**  ***proGhPP2AA2-ΔG2-*R**  ***proGhPP2AA2-mG*-F**  ***proGhPP2AA2-mG*-R**  ***pHis-GhPP2AA2*-F**  ***pHis-GhPP2AA2*-R**  ***pHis-GhPP2AA2-ΔC*-F**  ***pHis-GhPP2AA2-ΔC*-R**  ***GhL1L1-LUC*-F**  ***GhL1L1-LUC-*R**  ***proPP2AA2-LUC*-F**  ***proPP2AA2*-LUC-R**  ***proGhPP2AA2-ΔC*-LUC-F**  ***proGhPP2AA2-ΔC*-LUC-R**  **GhPP2AA2 -MBP-F**  **GhPP2AA2 -MBP-R**  **GhPIN1-HL-BP-F**  **GhPIN1-HL-BP-R**  **GhPP2AA2-P3-F**  **GhPP2AA2-P2-R**  **GhPIN1-P1-F**  **GhPIN1-P4-R**  **GhJAZ-P3-F**  **GhJAZ-P2-R**  **GhCIPK-P1-F**  **GhCIPK-P4-R**  **GhPIN1-HL-P4-R** | TCCATGGCTACCGTAAACTCCC  CCACTGCAGCCTGTGATGAGC  TTGTCACTGAAGCGGGAAGG  CGATACCGTAAAGCACGAGGAA  TCCATGGCTACCGTAAACTCCC  TTAGCAATGGGCATGAAACGG  AGCAGGGGATTTTCGATGG  TCAAAGTGATTAGCCAAAGAAGC  CGTGGTGATGAGTTCAGTCGT  GATGTTACCGGCGTTGCTG  GCCTGCAATTCCCATACAACCA  GCATCTACTTGTGTTGAAGCCCC  CCCACACCCATTACACAGACAGT  TGTCTCGGCCTCGGCACTT  GATCCCATCCGTTGGCCTAAT  CAAATCAGGGTGTTGCGTAAGC  AACCAACATCAACACCTCCTCC  GGCCTTCAAGCACCACAGTC  AGAGGACCTACTCCAAGGCCATC  CTCCTTAGGCTGCTGCTGTGG  GACCGACACCCAGACCATCAA  CCTTACCACCACCATCTTCAACC  AGGAAGTTGAAGGGAAGGGAG  TTGGAAGATGAAGGCGAAGAC  AAAGGCGAAGAACAGCATCAC  TCGGTGTTGCCATAAAGAAGC  TCTGGAGGGTCAAGACCGTCG  GCATGGATAATTTCACCGCCG  TGTTGGTGCTGGACCTTCTGG  ATCGGGTGAATGGGGTTTGAA  GAAGGCATTCCACCTGACCAAC  CTTGACCTTCTTCTTCTTGTGCTTG  GACGGAGTTTACCTTTTTGATTTTG  CAAAGTTGGATAAGACGAAACCCTA  CTGAGGTACAGTGCAGGGAAGA  CAAAGTTGGATAAGACGAAACCCTA  GACGGAGTTTACCTTTTTGATTTTG  GGGTTGAGACGGAAAATTGATCAACCCACCATTTTCTGACAAATGC  GCATTTGTCAGAAAATGGTGGGTTGATCAATTTTCCGTCTCAACCC  CAAAGTTGGATAAGACGAAACCCTA  AATCAAAAGAGTAACCAAGGTATTTTAAG  CTTAAAATACCTTGGTTACTCTTTTGATT  ATTCCCGGGGAGCTCACGCGTGACGGAGTTTACCTTTTTGATTTTG  ATAATGCCAGGAATTTCTAGACAAAGTTGGATAAGACGAAACCCTA  ATTCCCGGGGAGCTCACGCGTCTGAGGTACAGTGCAGGGAAGA  ATAATGCCAGGAATTTCTAGACAAAGTTGGATAAGACGAAACCCTA  CGCTCTAGAACTAGTGGATCCATGGAACGTGGAGGCTTCCATGG  CTTGATATCGAATTCCTGCAGTCATTTATGCTGCTGGGCATATG  CTTGATATCGAATTCCTGCAGGACGGAGTTTACCTTTTTGATTTTG  CGCTCTAGAACTAGTGGATCCCAAAGTTGGATAAGACGAAACCCTA  CTTGATATCGAATTCCTGCAGCTGAGGTACAGTGCAGGGAAGA  CGCTCTAGAACTAGTGGATCCCAAAGTTGGATAAGACGAAACCCTA  AAGGATTTCAGAATTCATGTCAATGATTGAAGAGCCTTTG  GCAGGTCGACTCTAGACTAAGACATCATGACCTGATTGCATG  GGGGACAAGTTTGTACAAAAAAGCAGGCTCCATGATCACTTTAACAGATTTTTACC  GGGGACCACTTTGTACAAGAAAGCTGGGTCTTACCACCACCATCTTCAACCT  GGGGACAACTTTGTATAATAAAGTTGGAATGTCAATGATTGAAGAGCCTTTG  GGGGACCACTTTGTACAAGAAAGCTGGGTCTAAGACATCATGACCTGATTGCATG  GGGGACAAGTTTGTACAAAAAAGCAGGCTTAATGTCGCTCGACGGTCGGC  GGGGACAACTTTGTATAGAAAAGTTGGGTTTAATTTTGAGGTGATGCCATAA  ACAACTTTGTATAATAAAGTTGGAATGAATATGTCGTGTTCACCGG  ACCACTTTGTACAAGAAAGCTGGGTGCTACGGAGATTGAGCAGCCAA  GGGGACAAGTTTGTACAAAAAAGCAGGCTTAATGGCGGACAAAGCTAAAACC  GGGGACAACTTTGTATAGAAAAGTTGGGTGAGCCACAGTCGAGTTCTCGGG  GGGGACAACTTTGTATAGAAAAGTTGGGTTTACTTACCACCACCATCTTCAAC |  | Northern blotting  Southern blotting  qRT-PCR  qRT-PCR  qRT-PCR  qRT-PCR  qRT-PCR  qRT-PCR  qRT-PCR  qRT-PCR  qRT-PCR  qRT-PCR  qRT-PCR  qRT-PCR  qRT-PCR  qRT-PCR  *proGhPP2AA2* cloning  *proGhPP2AA2-ΔC* cloning  *proGhPP2AA2-ΔC* cloning  *proGhPP2AA2-ΔC* cloning  *proGhPP2AA2-mG* cloning  Y1H for *proGhPP2AA2*, *proGhPP2AA2-mG* and *proGhPP2AA2-ΔG*  Y1H for *proGhPP2AA2-ΔC*  LUC for GhL1L1  LUC for *proGhPP2AA2*, *proGhPP2AA2-mG* and *proGhPP2AA2-ΔG*  LUC for *proGhPP2AA2-ΔC*  GST fused protein for pull-down  GST fused protein for pull-down  BiFC, FRET  BiFC, FRET  BiFC  BiFC  BiFC, FRET |
